# Navigating the Transition to RNA-Based Influenza Vaccines: A State-of-the-Art Review

**DOI:** 10.3390/vaccines14100874

**Published:** 2026-10-02

**Authors:** Antonios-Periklis Panagiotopoulos, Deny Tsakri, Alexandros Laskaris, Chrysa Saldari, Kyriaki Ranellou, Ilias Mariolis, Athanasios Tsakris, Cleo Anastassopoulou

**Affiliations:** 1 Department of Microbiology, Medical School, National and Kapodistrian University of Athens, 75 Mikras Asias Street, 115 27 Athens, Greece; anpanagiotop@med.uoa.gr (A.-P.P.); dtsakri@med.uoa.gr (D.T.); alexalas@med.uoa.gr (A.L.); chrysasaldari@gmail.com (C.S.); ranellouk@med.uoa.gr (K.R.); imariolis@med.uoa.gr (I.M.); cleoa@med.uoa.gr (C.A.); 2 First Department of Critical Care Medicine & Pulmonary Services, School of Medicine, National and Kapodistrian University of Athens, Evangelismos Hospital, 106 76 Athens, Greece; 3 2nd Department of Medicine and Laboratory, School of Medicine, National and Kapodistrian University of Athens, General Hospital of Athens “Hippokration”, 115 27 Athens, Greece

**Keywords:** influenza, H5N1, mRNA vaccines, saRNA, pandemic preparedness, clinical trials

## Abstract

Seasonal influenza and the ongoing emergence of zoonotic influenza viruses, including highly pathogenic avian influenza A (H5N1), remain substantial global public health threats, underscoring the need for rapidly adaptable vaccine platforms. This state-of-the-art review critically evaluates the clinical development of RNA-based influenza vaccines through a comprehensive assessment of ClinicalTrials.gov records and peer-reviewed literature. Candidates were examined across four domains: seasonal influenza vaccines, pre-pandemic influenza vaccines, combination respiratory vaccines, and self-amplifying RNA (saRNA) platforms. Clinical evidence indicates that mRNA influenza vaccines consistently elicit robust humoral immune responses and, in late-stage trials, have demonstrated improved relative efficacy against influenza A compared with conventional influenza vaccines. Reactogenicity is generally more frequent with mRNA vaccines, particularly with respect to transient mild-to-moderate systemic adverse events. However, serious adverse events remain uncommon and broadly comparable to those observed with licensed comparator vaccines. Pre-pandemic and multicomponent mRNA vaccine candidates similarly demonstrate strong immunogenicity, supporting the potential of RNA platforms for both seasonal vaccination and pandemic preparedness. Clinical development of saRNA influenza vaccines has progressed through early-phase trials, although publicly available clinical data remain limited. Regulatory milestones in 2026, including U.S. approval of mRNA-1010 (mFlusiva), the first approved mRNA-based seasonal influenza vaccine, and European Union authorization of mRNA-1083 (mCOMBRIAX), an mRNA-based influenza-COVID-19 combination vaccine, represent important steps in the clinical transition and implementation of RNA-based influenza vaccination.

## 1. Introduction

Seasonal influenza and emerging pandemic threats continue to impose a substantial global health burden, causing approximately one billion infections annually and up to 650,000 respiratory deaths worldwide [1]. Despite the availability of licensed vaccines, the continued reliance on conventional egg-based manufacturing leaves seasonal influenza vaccination vulnerable to rapidly evolving viral strains. Lengthy production timelines can delay vaccine updates and facilitate the emergence of egg-adapted mutations that may reduce antigenic match and clinical effectiveness [2,3]. This inflexibility represents a major challenge for pandemic preparedness, particularly as highly pathogenic avian influenza (HPAI) A viruses, such as H5N1, continue to spread among birds and mammals, increasing concerns regarding their potential to cause human outbreaks [4].

The clinical viability of mRNA vaccine platforms as rapid and adaptable countermeasures was demonstrated during the COVID-19 pandemic, complementing other therapeutic approaches, including antivirals [5,6,7]. Nationwide surveillance over a four-year period further supported the long-term effectiveness of mRNA vaccination, reporting 25% lower risk of all-cause mortality and 74% lower risk of death attributable to severe COVID-19 among vaccinated individuals [8]. Clinical studies reported baseline efficacy of 87–92% against symptomatic disease caused by pre-Delta SARS-CoV-2 variants for mRNA vaccines, compared with 76% for the traditional vaccine comparator [9,10]. However, because vaccine-induced immunity naturally wanes over time, booster doses are often required to maintain protection, particularly among older adults and individuals with underlying comorbidities [11,12].

The application of mRNA technology to influenza could address several limitations of conventional vaccine manufacturing by enabling substantially faster incorporation of updated viral sequences as new strains and threats emerge [2,13]. This structural flexibility may also facilitate the incorporation of antigens targeting multiple circulating respiratory viruses into a single vaccine formulation, potentially broadening protection while simplifying vaccination schedules [2,13]. In addition, mRNA vaccines combine the immunostimulatory properties of the RNA platform with those of lipid nanoparticle delivery systems, potentially reducing or eliminating the need for conventional external adjuvants [14]. Together, these manufacturing and immunological advantages position mRNA platforms as promising candidates for transforming influenza vaccination.

Herein, the rapidly evolving landscape of mRNA-based influenza vaccination is examined, with particular emphasis on the transition from conventional vaccine technologies to mRNA platforms. By critically evaluating the current clinical trial landscape, this review highlights the translational potential of mRNA influenza vaccines while addressing the remaining scientific, clinical, manufacturing, and regulatory challenges that must be overcome to achieve effective and globally accessible protection against seasonal and pre-pandemic influenza threats.

## 2. Methods and Search Criteria

To characterize the clinical landscape of mRNA vaccines for influenza, a comprehensive search of clinical trial registries and the peer-reviewed literature was conducted. The primary search was performed using the expert search function of the ClinicalTrials.gov registry, with the term “mRNA influenza vaccine” used to identify potentially relevant clinical trials. Additional trials were identified through supplementary searches using alternative keyword combinations to maximize the sensitivity of trial identification. For each eligible trial, corresponding publications were identified through PubMed searches using the associated ClinicalTrials.gov identifier and/or the trial title. A complementary search of the United Kingdom and Australian–New Zealand clinical trial registries was conducted to identify additional relevant clinical trials.

To identify published clinical outcomes associated with mRNA influenza vaccination, a parallel search of PubMed was conducted on 5 August 2026, using the following search query: (“mRNA”[Title/Abstract] OR “messenger RNA”[Title/Abstract] OR “RNA”[Title/Abstract]) AND (“Influenza, Human”[Mesh] OR “influenza”[Title/Abstract] OR “flu”[Title/Abstract]) AND (“Vaccines”[Mesh] OR “vaccine*”[Title/Abstract] OR “immunization”[Title/Abstract]) AND (“clinical trial*”[Title/Abstract] OR “randomized”[Title/Abstract]). The search was restricted to publications dated from 2020 through 5 August 2026, thereby focusing the analysis on the period following the emergence of SARS-CoV-2 and the subsequent acceleration of mRNA vaccine development.

## 3. The Current mRNA Influenza Vaccine Landscape

**Figure 1:**
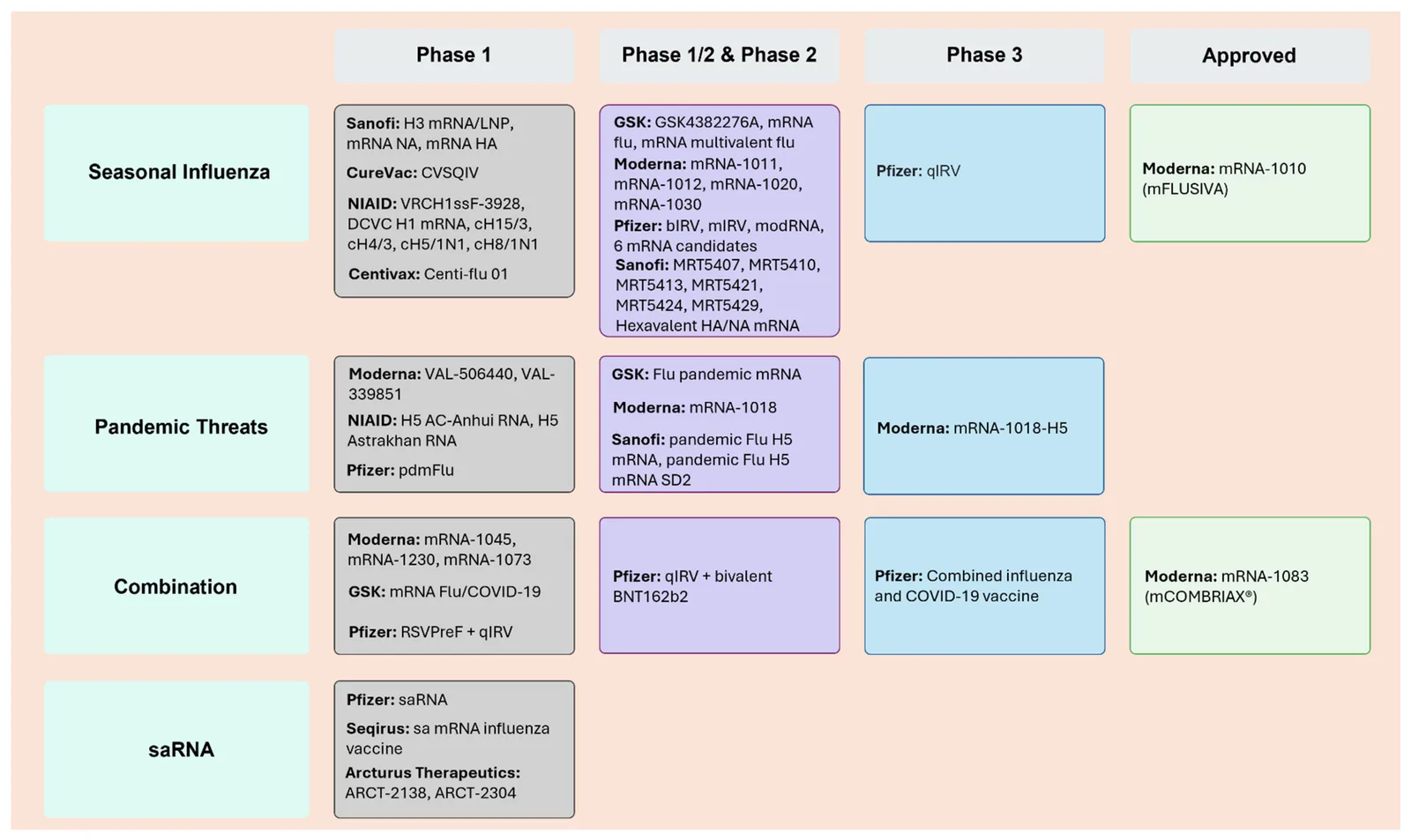
Clinical trial landscape of mRNA influenza vaccines. Note: For vaccine candidates evaluated in multiple clinical trials, only the most advanced phase of development is presented.

The current clinical development landscape of mRNA-based influenza vaccines is reviewed according to four target categories: (1) seasonal influenza, (2) influenza subtypes with pandemic potential, (3) combination vaccines targeting influenza and other respiratory viruses and (4) self-amplifying RNA (saRNA) vaccine candidates. Within each category, the major developers and manufacturers, vaccine candidates, clinical-trial phases and key development milestones are described. A comprehensive overview of the clinical development landscape of mRNA-based influenza vaccines, categorized by development phase and vaccine target, is presented in Figure 1.

### 3.1. Seasonal Influenza Pipeline

Seasonal influenza imposes a consistent global health burden, with approximately one billion cases occurring annually, including 3–5 million cases of severe illness [1]. Regional surveillance reflects this considerable morbidity. In the European Union/European Economic Area, seasonal influenza is associated with up to 50 million symptomatic cases and approximately 15,000–70,000 deaths annually [15]. In the United States, CDC estimates indicate that influenza caused 9.4–51 million illnesses, 120,000–710,000 hospitalizations and 6300–52,000 deaths annually between 2010 and 2025, excluding the 2020–2021 season because of minimal influenza activity [16]. The year-to-year variability in disease burden reflects differences in circulating strains, population immunity, vaccine uptake and vaccine effectiveness, underscoring the continued need for improved influenza vaccines with broader and more consistent protection [16,17].

#### 3.1.1. Moderna’s mRNA-1010

On 5 August 2026, the U.S. Food and Drug Administration approved mRNA-1010 (mFLUSIVA, ModernaTX, Inc., Cambridge, MA, USA), the first mRNA-based seasonal influenza vaccine licensed in the United States, marking an important regulatory milestone for the application of the mRNA platform beyond COVID-19 [18]. The vaccine is indicated for adults aged 50 years and older, with traditional approval for individuals aged 50–64 years and accelerated approval for those aged ≥65 years [18]. The regulatory decision was supported by a clinical development program encompassing early Phase 1/2 dose-selection, safety, and immunogenicity studies, subsequent Phase 3 immunogenicity and safety studies, and the pivotal P304 efficacy trial [19,20,21,22,23,24,25,26,27,28,29,30,31,32,33,34].

In the pivotal Phase 3 P304 trial, 40,703 adults aged ≥50 years received mRNA-1010 or a licensed standard-dose influenza vaccine. During a median follow-up of 181 days, RT-PCR-confirmed, protocol-defined influenza-like illness occurred in 2.0% of mRNA-1010 recipients compared with 2.8% of standard-dose vaccine recipients, corresponding to a relative vaccine efficacy of 26.6% (95% CI, 16.7–35.4) and meeting the prespecified criteria for noninferiority, superiority and higher-level superiority [28]. The reported relative vaccine efficacy was 27.4% among adults aged ≥65 years [28]. These findings demonstrate superiority over the standard-dose comparator, although they should not be interpreted as a direct efficacy comparison with high-dose or adjuvanted influenza vaccines, because those products are not the comparator in the pivotal efficacy study [28]. For adults ≥65 years, the FDA’s accelerated-approval decision incorporated both the clinical efficacy subgroup data and immunogenicity findings from studies comparing mRNA-1010 with standard- and high-dose vaccines [18,28].

Reactogenicity was more frequent with mRNA-1010 than with the standard-dose comparator. Injection-site pain occurred in 65.8% of mRNA-1010 recipients versus 29.8% of controls, while fatigue, headache and myalgia occurred in 45.1%, 37.8% and 35.4% of recipients, respectively, compared with 20.3%, 18.0% and 11.6% in the comparator group. Most solicited reactions were mild to moderate and transient [28]. Serious adverse events (SAEs) occurred in 2.2% of mRNA-1010 recipients and 1.9% of standard-dose vaccine recipients; three events in the mRNA-1010 group and two in the comparator group were considered vaccine-related by investigators. No vaccine-related deaths were reported [28]. Earlier Phase 1/2 studies likewise reported predominantly grade 1–2 reactogenicity and no vaccine-related SAEs or deaths, supporting a generally consistent safety profile across the development program [19,20,21,22,23,24,25,26,27].

As a condition of accelerated approval in adults aged ≥65 years, the FDA required a Phase 4 postmarketing study to verify and further characterize clinical benefit. The planned study, involving approximately 800,000 adults aged ≥65 years across two influenza seasons, will evaluate relative vaccine effectiveness and provide descriptive strain-specific analyses, including for influenza B/Victoria. Residual uncertainty regarding B/Victoria effectiveness remains because the B/Victoria-specific correlate-of-risk analysis did not demonstrate a statistically significant correlation between HAI titers and RT-PCR-confirmed, protocol-defined influenza-like illness caused by B/Victoria [18]. Accordingly, the FDA requested an exploratory correlate-of-risk analysis using microneutralization (MN) antibody titers to determine whether MN may provide a more appropriate surrogate immune marker for mRNA-1010; however, whether MN is a superior or validated surrogate remains to be established.

#### 3.1.2. Moderna’s Next Generation Formulations for Seasonal Influenza

The evolution of the influenza virus landscape has also driven development of next-generation mRNA formulations. In particular, the continued absence of confirmed naturally circulating influenza B/Yamagata viruses since March 2020 has led the World Health Organization (WHO) to conclude that inclusion of the B/Yamagata antigen in seasonal vaccines is no longer warranted and to encourage the transition from quadrivalent to trivalent formulations [2,35,36,37,38]. Rather than treating B/Yamagata as definitively extinct, this should be understood as an epidemiological absence based on global surveillance, with WHO recommending that the component be removed as soon as possible [36,37,38,39].

Against this background, Moderna has evaluated mRNA-1011.1, mRNA-1011.2 and mRNA-1012 as next-generation formulations designed to broaden coverage against antigenically distinct A/H3N2 viruses while adapting the composition to the reduced relevance of B/Yamagata [40,41]. In a Phase 1/2 randomized trial in adults aged 50–75 years, mRNA-1011.1 incorporated an additional A/H3N2 strain, mRNA-1011.2 replaced the B/Yamagata component with an additional A/H3N2 strain, and mRNA-1012 incorporated two additional A/H3N2 strains in place of B/Yamagata [40]. All three candidates generated robust antibody responses against vaccine-matched influenza strains and higher responses against the additional A/H3N2 strains, without reducing responses against A/H1N1 or influenza B/Victoria [40]. Solicited adverse reactions were broadly similar across candidate and mRNA-1010 control groups, and no vaccine-related SAEs or deaths were reported [40,41].

A complementary strategy is represented by Moderna’s mRNA-1020 and mRNA-1030 candidates, which encode both hemagglutinin (HA) and neuraminidase (NA) rather than targeting HA alone [42,43]. In a Phase 1/2 study, the two candidates used different HA:NA ratios and generated HAI responses comparable to those induced by HA-only mRNA and recombinant vaccine comparators while additionally inducing substantial NA-specific antibody responses [42,43]. HA- and NA-specific antibody titers remained above baseline through day 181, demonstrating persistence of the broader immune response [42,43]. Reactogenicity was primarily characterized by injection-site pain and fatigue, with no vaccine-related SAEs or significant safety concerns through the 181-day follow-up period [42,43]. These findings provide proof of concept for simultaneously targeting HA and NA, although clinical protection has not yet been established for these candidates in an efficacy trial.

#### 3.1.3. GSK’s mRNA Influenza Program Based on CureVac’s Technology

CureVac and GlaxoSmithKline (GSK) collaborated on an investigational mRNA-based seasonal influenza vaccine program. Their main candidate was a quadrivalent mRNA seasonal influenza vaccine candidate that was based on CureVac’s second-generation mRNA backbone and designed to encode antigens corresponding to the WHO-recommended seasonal influenza strains covering influenza A (H1N1, H3N2) and B (B/Yamagata and B/Victoria) [44].

The initial Phase 1 evaluation of the monovalent GSK4382276A candidate demonstrated an acceptable safety and reactogenicity profile characterized primarily by transient, dose-dependent local and systemic reactions [45,46]. The vaccine induced robust HAI antibody responses, strong expansion of memory B cells, and Th1-biased CD4+ T-cell responses, with higher-dose groups showing stronger immune responses than licensed comparator vaccines [45,46]. Early data showed strong immune responses against influenza A strains, while responses against influenza B were less robust, prompting further optimization [47]. GSK and CureVac restructured their partnership in July 2024. Full global rights to develop, manufacture and commercialize their mRNA influenza vaccine candidates, including the seasonal influenza program, were acquired by GSK [48]. CureVac retained an economic interest through milestone payments and royalties.

Subsequent Phase 1/2 and Phase 2a studies of multivalent formulations enrolled more than 2000 adults in total. These studies evaluated younger and older adults and demonstrated strong immune responses against influenza A and B strains, with an overall acceptable reactogenicity and safety profile [49,50]. These formulations maintained acceptable reactogenicity profiles, with adverse events primarily consisting of transient injection-site pain and fatigue and no vaccine-related SAEs reported, supporting the continued clinical development of the program toward Phase 3 [50,51]. GSK subsequently evaluated additional multivalent mRNA formulations in Phase 2a studies to further characterize immunogenicity, safety, and dosing in younger and older adults [52,53]. GSK’s CureVac-derived mRNA-based seasonal influenza program (not to be confused with GSK’s existing non-mRNA influenza products such as Fluarix) remains investigational at present.

#### 3.1.4. NIAID’s Early-Stage mRNA Platforms

The National Institute of Allergy and Infectious Diseases (NIAID) is investigating alternative, early-stage mRNA platforms through targeted Phase 1 dose-escalation studies in younger adults aged 18–49 years. In the absence of reported clinical efficacy results, these exploratory trials primarily aimed to characterize safety and reactogenicity, evaluate dose levels, and assess preliminary immunogenicity, including comparisons with licensed quadrivalent inactivated influenza vaccine (IIV4) where applicable. Key candidates include VRC H1ssF-3928, a single-dose HA stem-based mRNA-LNP vaccine designed to induce anti-stalk serum antibodies (NCT05755620), and DCVC H1, an HA-based mRNA-LNP candidate evaluated as a two-dose primary series (NCT05945485) [54,55]. A newly initiated Phase 1 dose-ranging trial evaluates a series of chimeric HA mRNA candidates, specifically cH8/1, cH5/1, cH15/3 and cH4/3, in healthy adults aged 18 to 59 years [56]. These candidates are designed to target influenza A Group 1 and Group 2 viruses by inducing anti-HA stalk antibodies [56].

#### 3.1.5. Pfizer’s Seasonal Influenza mRNA Program

Pfizer has advanced its nucleoside-modified messenger RNA (modRNA) seasonal influenza vaccine platform through Phase 1/2 and pivotal Phase 3 clinical development. The first-generation quadrivalent modRNA influenza vaccine candidate (qIRV) encodes the HA proteins of four seasonally recommended influenza strains, comprising two influenza A and two influenza B strains [57,58]. The Phase 1/2 program enrolled 1158 healthy adults aged 18–85 years and evaluated monovalent, bivalent, and quadrivalent formulations across different dose levels [57,58]. The study supported further evaluation of qIRV at doses of 30 μg for younger adults aged 18–64 years and 60 μg for older adults [58]. In participants aged 65–85 years, qIRV generally elicited higher HAI antibody responses against influenza A strains than the licensed quadrivalent influenza vaccine (QIV), whereas responses against influenza B strains were generally lower [58]. In contrast, qIRV induced overall higher CD4+ and CD8+ T-cell responses against all four vaccine-encoded strains, suggesting that the cellular immune response may provide an additional immunological advantage not fully captured by HAI antibody titres [58].

The safety and tolerability profile of qIRV was considered acceptable, with injection-site pain representing the most frequently reported local reaction [58]. Solicited local and systemic reactions were generally mild to moderate and occurred more frequently with qIRV than with licensed QIV, with some evidence of dose-related increases in reactogenicity. No new major safety concerns were identified during the Phase 1/2 program [57,58].

These findings supported progression to a pivotal Phase 3 efficacy trial (NCT05540522) in adults aged 18–64 years [59]. The randomized trial enrolled 18,476 participants and compared qIRV with a licensed quadrivalent inactivated influenza vaccine [59,60]. The relative efficacy of qIRV versus the licensed vaccine against influenza-like illness associated with laboratory-confirmed influenza was 34.5% (95% CI, 7.4–53.9%), meeting prespecified criteria for both noninferiority and superiority [59,60]. However, the observed clinical efficacy was driven predominantly by influenza A, as almost no influenza B cases occurred during the study period [60]. Consistent with the Phase 1/2 findings, HAI antibody responses were noninferior for influenza A strains but not for influenza B strains [60]. Reactogenicity was also more frequent with qIRV, although most reported reactions were mild or moderate [60].

Taken together, these findings demonstrate the potential of the modRNA platform to enhance immune responses and clinical protection against influenza A while also highlighting the persistent challenge of achieving robust and consistent immune responses against influenza B. In response to this limitation, Pfizer subsequently evaluated second-generation modRNA influenza vaccine candidates in a Phase 2 study (NCT06436703), with the aim of improving influenza B immunogenicity and potentially broadening protection [61]. In a Phase 2a human challenge trial, healthy adults who received monovalent or bivalent modRNA formulations demonstrated 100% (95% CI, 75.2–100.0%) absolute vaccine efficacy against symptomatic infection following exposure to an influenza A/H1N1 strain when compared to an unvaccinated group, whereas the licensed quadrivalent vaccine demonstrated an absolute efficacy of 84.5% (95% CI, 43.4–96.0%) [62,63]. The mRNA vaccines also reduced the peak viral load more effectively than the conventional vaccine, and no serious adverse events were reported among the participants [62,63]. A subsequent Phase 2a challenge study evaluated trivalent and quadrivalent modRNA candidates specifically against exposure to the influenza B/Connecticut/1/21 virus (ISRCTN13559330). Although the clinical phase of this trial has ended, efficacy and safety outcomes have not yet been published [64].

Pfizer is also evaluating six additional experimental mRNA influenza vaccine candidates in an ongoing randomized, double-blind Phase 2 trial (NCT07431853), designed to assess their safety, tolerability, and immunogenicity in adults aged 18 years and older [65].

As of August 2026, Pfizer’s seasonal influenza mRNA program remains investigational. Although the first-generation quadrivalent modRNA influenza vaccine (qIRV) demonstrated superior relative efficacy versus a licensed influenza vaccine in adults aged 18–64 years, Pfizer has not obtained regulatory approval for an mRNA-based seasonal influenza vaccine. Current development is focused on next-generation mRNA candidates intended to improve immunogenicity and broaden protection, with particular emphasis on achieving more robust and consistent responses against influenza B.

#### 3.1.6. Sanofi’s Seasonal Influenza mRNA Clinical Program and Subsequent Discontinuation

Sanofi, through Sanofi Pasteur and its mRNA platform derived in part from the 2021 acquisition of Translate Bio, conducted a broad clinical development program evaluating mRNA-based seasonal influenza vaccines targeting both HA and NA. Early clinical studies included a monovalent mRNA-NA candidate (NCT05426174) [66], monovalent HA candidates (NCT05829356 and NCT06118151) [67,68], and several quadrivalent HA-based candidates, including MRT5407 (NCT05553301), MRT5410 (NCT05624606), and MRT5413 (NCT05650554) [69,70,71]. These studies were generally Phase 1 or Phase 1/2 and evaluated safety and immunogenicity against licensed influenza-vaccine comparators and have subsequently been completed.

A later Phase 1/2 study, NCT06361875, evaluated three quadrivalent mRNA influenza vaccine candidates—MRT5421, MRT5424, and MRT5429—in 910 adults aged ≥18 years [72]. Investigational vaccines were administered as single intramuscular doses and compared with standard-dose or high-dose quadrivalent influenza vaccine (in adults aged ≥65 years), or quadrivalent recombinant influenza vaccine [72]. The study was completed on 9 June 2025. The available results indicate that by Day 29 post-vaccination, the mRNA candidates induced robust HAI antibody responses against influenza A strains, whereas the responses against influenza B viruses were weaker [72]. Injection site reactions and systemic adverse events occurred more frequently in the mRNA group compared to the controls [72]. SAEs remained rare across all mRNA candidates at rates below 3.3%, which was comparable to the rates observed in the conventional vaccine controls [72]. These findings are consistent with the principal development challenge encountered by many seasonal influenza mRNA programs: achieving sufficiently broad and consistent immunogenicity across both influenza A and B antigens while maintaining an acceptable reactogenicity profile.

Sanofi subsequently advanced a next-generation hexavalent mRNA influenza vaccine designed to combine trivalent mRNA-HA with trivalent mRNA-NA [73]. This approach was evaluated in a Phase 1/2 study comparing different HA+NA formulations with standard- and high-dose licensed influenza vaccines in adults aged ≥50 years [73].

It began in January 2025 and was completed on 22 May 2026. Nevertheless, Sanofi had already deprioritized its seasonal influenza mRNA vaccine program in January 2026 and discontinued the clinical development effort spanning monovalent HA/NA, quadrivalent HA, and hexavalent HA+NA candidates.

Overall, Sanofi’s program illustrates the broader challenge of translating the potential immunogenicity, antigenic flexibility and manufacturing advantages of mRNA technology into a competitive seasonal influenza vaccine. Despite progression through multiple Phase 1 and Phase 1/2 studies, the mRNA program did not advance to Phase 3 efficacy testing or regulatory submission. As of August 2026, Sanofi therefore has no approved seasonal influenza mRNA vaccine and no active late-stage seasonal influenza mRNA program, while continuing to maintain a substantial conventional influenza vaccine portfolio.

#### 3.1.7. Centivax’s Universal mRNA Influenza Vaccine Program

Centivax has initiated a Phase 1a/b randomized, double-blind, placebo-controlled clinical trial to evaluate Centi-Flu 01, an investigational universal mRNA influenza vaccine candidate [74,75]. Centi-Flu 01 targets highly conserved and mutation-resistant regions of the influenza virus to generate broad humoral and cellular immune responses against multiple seasonal and pre-pandemic strains [75,76]. According to the developer, it will not need annual reformulations [75,76]. The trial assesses immunogenicity by measuring HAI antibody titers against a panel of more than 20 diverse influenza strains, with a Phase 2 trial scheduled to begin in early 2027 [74,75,76].

The characteristics and current clinical development status of seasonal mRNA influenza vaccine candidates are summarized in Table 1.

### 3.2. Vaccines Against Influenza Subtypes with Pandemic Potential

Clinical management of H5N1 avian influenza remains challenging because current treatment relies primarily on broad-spectrum influenza antivirals, while no H5N1-specific therapeutic has been licensed [77,78]. Although seasonal influenza vaccination can induce some cross-reactive immune responses, recent evidence indicates that it does not reliably generate neutralizing antibodies against contemporary highly pathogenic avian influenza A(H5N1) clade 2.3.4.4b viruses and therefore cannot substitute for a strain-matched pandemic vaccine strategy [79,80]. The adaptability of mRNA technology, together with its established lipid-nanoparticle delivery platform and potential for rapid sequence modification, therefore, provides an attractive approach to pre-pandemic vaccine development and pandemic preparedness.

#### 3.2.1. Early mRNA Vaccine Candidates by Moderna

The earliest clinical evaluation of mRNA vaccines against avian influenza viruses with pandemic potential began with Moderna’s VAL-506440 and VAL-339851, which target H10N8 and H7N9, respectively [81,82,83]. In randomized Phase 1 trials, both unadjuvanted lipid nanoparticle formulations exhibited favorable tolerability profiles, with no vaccine-associated SAEs reported [81]. Regarding immunogenicity, the vaccines elicited robust HAI responses that maintained notable seropositivity for up to six months post-vaccination [81]. The H10N8 candidate produced particularly strong responses at higher doses, with persistence of HAI antibodies observed during follow-up, while the H7N9 candidate similarly demonstrated induction of HAI and microneutralization antibodies [81]. These studies provided early clinical evidence that an mRNA platform could generate functional antibody responses against influenza subtypes that had not previously circulated widely in humans.

#### 3.2.2. Moderna’s mRNA-1018

Building on this experience, the next-generation candidate mRNA-1018 (ModernaTX) was subsequently evaluated in a large Phase 1/2 dose-ranging study [84,85,86]. This trial assessed five distinct candidates divided into formulations H5N8, H5-only, H7N9 and H7-only formulations in Part A, and an H5-only-CG formulation in Part B [85]. A total of 1499 participants received at least one dose across the two study parts. The H5N8 and H7N9 candidates were administered at 25, 50 and 100 μg, whereas the H5-only, H7-only and H5-only-CG candidates were evaluated at 12.5, 25 and 50 μg [84,85]. All candidates were administered as two-dose intramuscular series 21 days apart [84,85].

The mRNA-1018 candidates demonstrated an overall favorable safety profile. Solicited local and systemic adverse reactions were predominantly grade 1 or 2, with injection site pain, fatigue and headache among the most frequently reported events [85]. Serious adverse events occurred in 24 participants in Part A and four in Part B; one SAE, syncope in a participant receiving 12.5 μg of H5-only-CG, was considered related to vaccination [85]. Additionally, one event of acute thyroiditis that led to discontinuation (H5-only 50 µg group) and two cases of mild thrombocytopenia, one in the 12.5 μg H5-only group and one in the 25 μg H5-only-CG group, were also considered vaccine-related [85].

Immunogenicity was similarly encouraging. All five candidates generated functional immune responses detectable before administration of the second dose, with responses generally peaking one to three weeks thereafter and remaining detectable through the six-month follow-up period [85]. H5-based candidates generated stronger strain-specific HAI responses than their H7-based counterparts, while antibody responses generally increased with increasing mRNA dose [85]. In the H5-only-CG arm, day-43 HAI geometric mean titers increased from 203.9 at 12.5 μg to 340.9 at 50 μg, while HAI seroconversion rates ranged from 94.6% to 100% [85]. In a complementary analysis of the H5-A/chicken/Ghana/2021 formulation, 97.8% of participants achieved HAI titers ≥ 1:40 three weeks after the second dose, a threshold conventionally used as a seroprotective correlate, while 70.6% retained titers at or above this threshold six months after vaccination [85]. These findings indicate rapid and durable humoral immunogenicity, although they should not be interpreted as direct evidence of clinical protection against H5N1 infection in humans.

A Phase 3 clinical trial initiated in 2026 is currently underway, enrolling approximately 4045 participants focusing on the evaluation of Moderna’s mRNA-1018-H5 pandemic influenza vaccine [87].

#### 3.2.3. Pfizer’s Pipeline of mRNA Vaccines Against Pre-Pandemic Influenza Threats

The broader development landscape has increasingly focused on highly pathogenic avian influenza A(H5N1) clade 2.3.4.4b [88]. Pfizer’s pre-pandemic influenza modRNA vaccine (pdmFlu; PF-07985819) encodes the HA of A/Astrakhan/3212/2020 (H5N8), a clade 2.3.4.4b prototype strain [89]. WHO preclinical studies in ferrets demonstrated strong neutralizing antibody responses and robust protection against lethal challenge with contemporary clade 2.3.4.4b H5N1 viruses, accompanied by reduced viral replication and, importantly, reduced virus shedding and onward transmission in a direct-contact model [89]. Because human H5N1 infections are sporadic and severe, establishing field efficacy in humans is logistically and ethically challenging, rendering preclinical animal models essential for evaluating protective efficacy [89]. Nevertheless, although these findings are highly relevant to pandemic preparedness, evidence from animal models cannot by itself establish vaccine efficacy in humans.

Pfizer subsequently evaluated pdmFlu in a randomized Phase 1 clinical trial (NCT06179446) involving healthy adults aged 18–49 and 65–84 years [90,91]. The trial enrolled 157 participants and compared pdmFlu with placebo and licensed seasonal influenza vaccine controls [90]. The study was completed in September 2025; however, as of August 2026, clinical results have not been posted on ClinicalTrials.gov [91]. Earlier interim analyses reported increased antibody responses against clade 2.3.4.4b H5 viruses, supporting continued clinical investigation of the platform.

#### 3.2.4. Other Developers’ mRNA Vaccine Candidates Against Avian Influenza Viruses with Pandemic Potential

Several other developers, including GSK, the National Institute of Allergy and Infectious Diseases (NIAID) and Sanofi, have completed concurrent Phase 1 and Phase 1/2 dose escalation trials for similar H5 mRNA formulations [88]. These trials utilized observation windows ranging from six to thirteen months, but no clinical results have been publicly posted to date, at the time of writing this article in August 2026 [92,93,94]. Meanwhile, an additional Phase 1/2 study for Sanofi’s second generation structurally designed (SD2) H5 mRNA vaccine against pandemic H5 influenza virus (pandemic flu H5 hemagglutinin (HA) mRNA SD2) is currently active, with completion estimated for December 2026 [95].

In summary, because human H5N1 infections are rare but associated with severe disease, conventional clinical trials designed to demonstrate direct protective efficacy are practically and ethically challenging, if not impossible, to conduct [78]. Consequently, the current clinical evidence supporting pre-pandemic H5N1 vaccines relies primarily on immunogenicity measures, such as vaccine-induced immune responses. Although these immunological correlates provide important evidence of potential protection, they cannot by themselves establish that vaccination will prevent severe disease following real-world exposure [78].

Clinical trials evaluating saRNA vaccine candidates against H5N1 are discussed in Section 3.4. Details of the clinical trials of mRNA vaccine candidates against avian influenza viruses with pandemic potential are presented in Table 2.

Collectively, these findings demonstrate substantial progress from the initial proof-of-concept studies of H10N8 and H7N9 mRNA vaccines toward H5-focused candidates specifically designed for contemporary pandemic threats. The most mature programs have now demonstrated favorable early safety profiles and strong HAI and neutralizing antibody responses, while preclinical data additionally suggest potential for reducing viral replication and transmission. Consequently, the principal value of these vaccines for pandemic preparedness currently lies in their combination of immunogenicity, platform adaptability and the potential for rapid sequence updating rather than demonstrated clinical efficacy against an H5N1 pre-pandemic strain.

### 3.3. Combination Vaccines Against Influenza and Other Respiratory Viruses

The post-pandemic landscape is characterized by the concurrent circulation of influenza, RSV and SARS-CoV-2, contributing to recurrent respiratory co-epidemics that can place substantial strain on healthcare systems [96,97]. Respiratory viral coinfection has been associated with greater disease severity and mortality compared with isolated viral infections [98]. Combination vaccines could therefore help address the changing epidemiology of respiratory viruses while reducing the number of annual vaccine administrations required for protection.

#### 3.3.1. mRNA-1073: Moderna’s First Multicomponent Candidate

Moderna’s first-generation multicomponent candidate mRNA-1073 combined the quadrivalent influenza vaccine candidate mRNA-1010 with the original mRNA-1273 COVID-19 vaccine, which encodes the full-length spike glycoprotein [99,100]. A Phase 1/2 clinical evaluation of mRNA-1073 in healthy adults demonstrated that a single dose elicited broad antibody responses comparable to those induced by the individual vaccine components [99,100]. mRNA-1073 was generally well tolerated, with mostly mild or moderate reactogenicity and no SAEs considered related to vaccination [99,100].

#### 3.3.2. mRNA-1083: The First Approved Combination Vaccine for Influenza and SARS-CoV-2

Moderna subsequently developed the next-generation candidate, mRNA-1083, which combines mRNA-1010 with the next-generation COVID-19 vaccine mRNA-1283 encoding only the specific N-terminal and receptor-binding domain of the spike glycoprotein [101,102,103,104,105,106]. While Phase 3 trials have not yet measured clinical efficacy, they utilized well established surrogate markers of protection to demonstrate that a single administration met all primary non-inferiority criteria [107]. mRNA-1083 successfully induced higher humoral immune responses against the target SARS-CoV-2 variant and seasonal influenza viruses (A/H1N1, A/H3N2, B/Victoria, and B/Yamagata) compared with licensed standard-of-care vaccines administered separately [107]. These robust immune responses were durable, maintaining antibody levels associated with protection against both respiratory pathogens throughout a six-month observation period [108].

The vaccine candidate also exhibited an acceptable safety profile characterized predominantly by mild-to-moderate transient AEs. The frequency of solicited reactions was 5.4% higher than standalone comparators in participants 65 years and older and 3.4% higher in those aged 50–65 years [99,107,108]. Investigators determined that no SAEs, or fatal events were considered related to vaccination [107,108].

mRNA-1083 (mCOMBRIAX^®^) was granted marketing authorization by the European Commission in April 2026 following a positive recommendation from the European Medicines Agency (EMA). mRNA-1083 is the first approved combination mRNA vaccine in Europe for prevention of seasonal influenza and COVID-19 in adults aged 50 years and older [109]. No approval by the FDA or active pending application currently exists for mCOMBRIAX^®^. Moderna voluntarily withdrew its U.S. Biologics License Application (BLA) to compile requested phase 3 influenza efficacy data [110].

#### 3.3.3. Pfizer-BioNTech’s Combination Vaccine Candidates

Pfizer-BioNTech has advanced its pipeline by combining its seasonal qIRV formulation with established, licensed vaccines targeting RSV (RSVpreF) and SARS-CoV-2 (BNT162b2) to develop multivalent candidates against co-circulating respiratory viruses [111,112]. These clinical trials have been completed, reporting promising early immunogenicity and tolerability profiles. Both dual formulations successfully elicited neutralizing antibodies against RSV and SARS-CoV-2 Omicron variants while preserving strong immune responses against all four seasonal influenza strains [111,112]. From a safety perspective, both combination candidates demonstrated an acceptable profile defined mostly by mild-to-moderate and transient reactogenicity, predominantly localized injection site pain and fatigue [111,112]. Severe complications were rare, with SAEs occurring at very low frequencies among the participants receiving the combination vaccines [111,112].

Subsequently, Pfizer-BioNTech advanced a phase 3 combined influenza and COVID-19 mRNA candidate [113,114]. Immunogenicity results met non-inferiority criteria for SARS-CoV-2, influenza A/H1N1 and influenza A/H3N2 compared to coadministered licensed vaccines [113]. However, the vaccine did not meet the prespecified non-inferiority criterion due to lower antibody titers for the influenza B/Victoria strain [113]. The safety profile remained acceptable, with most local and systemic reactions, including injection-site pain and fatigue, being mild or moderate and transient, though these events occurred more frequently following the combination vaccine than in the control groups [113]. Over a six-month monitoring period, SAEs were reported at a lower rate for participants receiving the combination vaccine (0.7–0.8%) compared to patients in the coadministered standalone comparator group (1.4%) [113,114]. Investigators determined that none of these reported SAEs were related to the study vaccination [113]. Six deaths occurred during the trial; none were considered related to vaccination [113].

Pfizer and BioNTech have completed two additional Phase 1/2 clinical trials evaluating their influenza and COVID-19 mRNA vaccine candidates in healthy adults aged 18 years and older [115,116]. NCT06821061 assessed the safety, tolerability, and immunogenicity of the modRNA vaccines when administered as standalone components or combined into a single injection [115]. Concurrently, NCT06683352 evaluated the standalone and combined mRNA candidates, utilizing an administration strategy of one injection administered in each upper arm [116]. Although both clinical studies have concluded, the final results have not yet been publicly posted [115,116].

#### 3.3.4. Other Early-Stage Combination Vaccine Candidates in Clinical Trials

Results have not yet been reported for several other combination candidates currently in clinical development. These include Moderna’s broader multicomponent formulations mRNA-1045 and mRNA-1230, which expand the combination approach to include respiratory syncytial virus (RSV) [117]. Similarly, GSK is evaluating its investigational seasonal influenza and SARS-CoV-2 combination mRNA vaccines through early clinical stages, with one Phase 1 trial officially completed (NCT06680375) [118] and another actively recruiting participants (NCT07464314) [119]. Table 3 summarizes the development phase and viral targets of the combination vaccine candidates.

### 3.4. Self-Amplifying RNA (saRNA) Vaccine Candidates

Self-amplifying RNA (saRNA) vaccines represent an extension of the conventional mRNA vaccine platform and differ primarily in their ability to amplify the delivered RNA intracellularly. Conventional mRNA vaccines consist of a non-replicating mRNA molecule encoding the target antigen. Following cellular uptake and release into the cytoplasm, the mRNA is directly translated by host ribosomes to produce the antigen, after which the RNA is progressively degraded. In contrast, saRNA vaccines are engineered RNA replicons, most commonly derived from positive-sense, single-stranded RNA viruses such as alphaviruses, in which the viral structural protein genes are substituted with a sequence encoding the target antigen [120,121]. Unlike conventional mRNA vaccines, these engineered replicons retain viral genes encoding the RNA-dependent RNA polymerase (RdRp) and associated replication machinery. Consequently, after delivery into the cytoplasm, the saRNA is first translated to produce the replication proteins, which subsequently amplify the antigen-encoding RNA and generate additional RNA templates for antigen production [122].

This difference in intracellular RNA metabolism has important implications for antigen expression and vaccine dosing. Whereas conventional mRNA vaccines rely on translation of the initially administered RNA molecules, saRNA can generate multiple copies of the antigen-encoding RNA within the cell, potentially resulting in higher and more prolonged antigen expression from a substantially lower administered RNA dose [120,121,122]. Thus, the principal potential advantage of saRNA is dose sparing, which may facilitate vaccine manufacturing and potentially improve the scalability of RNA-based vaccination. Prolonged antigen expression may also provide sustained antigen availability for the development of adaptive immune responses [120,121,122]. However, these potential advantages need to be balanced against the greater molecular complexity of saRNA and its interaction with intracellular innate immune sensing pathways.

An additional characteristic of saRNA is the generation of double-stranded RNA (dsRNA) intermediates during intracellular RNA replication. These intermediates can activate innate immune sensors and stimulate the production of type I interferons and proinflammatory cytokines [120,122]. This innate immune activation may contribute to the intrinsic adjuvant properties of saRNA and thereby enhance immune responses. However, excessive activation of innate immune pathways can also inhibit RNA translation and promote premature termination of antigen expression, potentially limiting the immunogenicity of the vaccine [121,122]. Therefore, an important challenge in saRNA vaccine development is achieving an appropriate balance between sufficient innate immune stimulation and efficient antigen production. To address this issue, different saRNA platforms have explored modifications to RNA sequence and chemistry, including the incorporation of modified nucleotides such as 5-methylcytidine, with the aim of reducing excessive innate immune activation while maintaining efficient and prolonged antigen expression [121].

Overall, compared with conventional mRNA vaccines, saRNA platforms have the potential to achieve comparable or enhanced antigen expression using substantially lower RNA doses and may provide more prolonged antigen production [120,121,122]. Nevertheless, these potential advantages should not be interpreted as established clinical superiority. Conventional mRNA vaccines have a considerably more extensive clinical evidence base, whereas clinical experience with saRNA vaccines remains comparatively limited, particularly for influenza vaccination. The clinical performance of saRNA may also depend on the specific replicon design, antigen, formulation, route of administration and degree of innate immune activation.

The current clinical landscape of saRNA vaccines against influenza comprises four candidates, as summarized in Table 4. All four candidates have officially reached completion, yet formal clinical outcomes and detailed safety results have not been reported in ClinicalTrials.gov or, to our knowledge, in the published scientific literature [62,123,124,125,126]. Two candidates are specifically designed to target pandemic H5N1 influenza viruses, whereas the remaining candidates target seasonal influenza strains [62,123,124,125,126]. Thus, although these candidates demonstrate increasing clinical interest in saRNA technology for influenza vaccination, the currently available evidence remains insufficient to establish the clinical efficacy, safety, durability of protection, or comparative advantages of saRNA vaccines over conventional mRNA vaccines in influenza.

## 4. Challenges Associated with the Use of mRNA Influenza Vaccines

### 4.1. Safety and Reactogenicity

The widespread administration of mRNA vaccines has been associated with a range of AEs, most of which are mild and transient, including fatigue and localized injection-site pain [5]. Although severe complications such as thrombosis, stroke, and cardiovascular events have been reported, their occurrence is rare [127,128]. In adolescents, clinical analyses estimate the incidence of myopericarditis following mRNA vaccination at approximately 43 cases per million doses (95% CI, 30.8–61.6) [129]. Similarly, anaphylaxis remains uncommon, occurring at approximately 13 cases per million administered doses (95% CI, 11.49–14.12) in pediatric and adolescent populations [130]. Importantly, large-scale studies indicate that the risk of several serious complications following SARS-CoV-2 infection is substantially greater than the corresponding risk following mRNA vaccination [131]. Comparisons with protein-subunit vaccines suggest that mRNA vaccines may be associated with a higher relative risk of certain transient adverse events, while differences in SAEs appear more limited and outcome-dependent [132,133].

An important challenge, however, is determining whether the AEs observed following COVID-19 mRNA vaccination are attributable primarily to the mRNA platform itself, to the vaccine-encoded spike protein, or to interactions between these components and host-specific factors. Although the overall safety profile of mRNA vaccines remains favorable, rare cases of myopericarditis represent a clinically important concern [134]. This adverse event occurs disproportionately among adolescent and young adult males, particularly following the second vaccine dose [134]. Recent surveillance data have also reported increased incidence rate ratios for myopericarditis, anaphylaxis, lymphadenopathy and appendicitis following the second dose of an mRNA vaccine [135]. Understanding the biological and immunological mechanisms underlying these age- and sex-associated differences is therefore an important priority for improving the safety of current vaccines and informing the development of next-generation mRNA platforms. Further investigation is needed to distinguish platform-specific effects from antigen-specific and host-dependent responses, particularly as mRNA technology is increasingly applied beyond COVID-19 vaccination.

### 4.2. Durability of Immune Response

The relatively rapid decline in circulating antibody responses represents a major challenge for the long-term clinical utility of mRNA vaccines against seasonal respiratory viruses [12]. Following the peak post-vaccination response, neutralizing antibody titers can drop significantly within approximately six months [136]. However, this reduction in circulating antibodies does not necessarily correspond to a complete loss of immune protection, as vaccination can generate immunological memory and durable protection against severe disease and hospitalization [12]. Consistent with broader immunological observations, cohort analyses of mRNA vaccination suggest that natural viral infection can induce antibody responses that are greater and more persistent over time than those generated by vaccination alone [137].

The decline in antibody-mediated protection has contributed to the use of booster vaccinations as a strategy for maintaining immunity over extended periods [138]. Although booster doses can effectively restore neutralizing antibody titers to high levels, their ability to substantially extend the persistence of circulating antibodies appears more limited [138]. Consequently, improving the intrinsic durability of vaccine-induced immunity remains an important objective for the development of next-generation mRNA platforms. Emerging approaches, including circular RNA, self-amplifying RNA and optimization of lipid nanoparticle (LNP) formulations, may extend antigen expression, improve antigen presentation and potentially enhance the magnitude and persistence of immune responses [139]. In addition, immunosenescence represents another critical challenge, as it is associated with a pronounced decline in both T-cell activation and neutralizing antibody titers, thereby accelerating the waning of vaccine-induced immunity in older adults [140].

Beyond the gradual decline in antibody titers over time, the long-term effectiveness of strain-matched influenza vaccines is inherently challenged by the continuous evolution of influenza viruses through antigenic drift and, in some influenza viruses, antigenic shift [2]. Addressing this antigenic diversity will require vaccine strategies that target highly conserved viral components, including the HA stalk, nucleoprotein, and matrix proteins [2]. mRNA platforms offer a promising approach for encoding such conserved antigens and may induce broader, cross-reactive cellular and humoral immune responses than those generated by strain-specific vaccines. By targeting viral regions that are less susceptible to antigenic variation, these approaches could potentially provide more durable and broader protection against genetically and antigenically divergent influenza viruses [2].

Such advances could reduce the frequency of booster administration and improve the practicality and long-term effectiveness of mRNA vaccination programs, while potentially increasing adherence to recommended immunization schedules [136].

### 4.3. Manufacturing and Logistical Challenges

The thermal instability of mRNA vaccines presents a significant barrier to their global distribution, as standard refrigeration may be insufficient to maintain clinical viability over extended periods [141]. The strict storage requirements conflict with the infrastructural realities of low- and middle-income countries (LMICs), where frequent electrical outages and a lack of specialized freezers hinder deployment efforts [142]. Cold-chain limitations also extend to transportation, as temperature fluctuations between central distribution hubs and remote rural clinics may compromise vaccine potency before administration. Although alternative cooling technologies, including solar-powered refrigerators, are being piloted, their large-scale implementation in LMICs remains constrained by financial and maintenance requirements [142]. Beyond logistical and infrastructural challenges, the high market prices of mRNA vaccines constitute a significant economic barrier to their equitable global distribution [143]. Consequently, the development of more thermostable, heat-resistant mRNA formulations remains a critical research priority, as it could reduce dependence on fragile cold-chain networks and facilitate more efficient vaccine distribution [141]. In parallel, strengthening local manufacturing capacity within LMICs could further reduce logistical barriers, enhance regional self-sufficiency and contribute to more equitable global access to mRNA vaccines [144].

### 4.4. Vaccine Hesitancy

Widespread vaccine hesitancy remains a significant barrier to the global acceptance and implementation of mRNA vaccines [145]. Public concerns commonly involve perceptions of limited safety, suboptimal effectiveness and distrust in regulatory authorities, with these concerns often amplified by online misinformation and conspiracy theories [145]. Vaccine hesitancy is not a uniform phenomenon, as individuals can be divided into distinct subgroups ranging from those who are uncertain or cautious to those who strongly reject vaccination [146]. Among older adults, additional factors, including a reduced perception of the severity of respiratory infections and confidence in their overall health, may further contribute to vaccine resistance [147]. Reducing the number of required injections through the development of multicomponent formulations could potentially address some practical and psychological barriers to vaccination, particularly as the perceived burden of receiving multiple vaccine doses is frequently cited by vaccine-hesitant individuals.

### 4.5. Influenza B Immunogenicity

Late-stage clinical trials indicate that mRNA-based influenza vaccines consistently elicit robust immune responses against influenza A subtypes but have more frequently failed to meet non-inferiority criteria for influenza B lineages. This comparatively reduced immunogenicity is not unique to mRNA platforms and has also been observed with conventional seasonal influenza vaccines [148,149]. Several overlapping biological and methodological factors may contribute to this disparity [148,149].

The intrinsic glycosylation patterns and conformational dynamics of influenza B HA may alter the accessibility of key epitopes and limit optimal antigen recognition and presentation following mRNA vaccination [148]. In addition, the comparatively slower evolutionary rate of influenza B viruses may contribute to immune imprinting, whereby repeated exposure to antigenically similar strains over an individual’s lifetime shapes subsequent antibody responses and potentially reduces the magnitude or breadth of responses to updated vaccine antigens [148,149]. Finally, assessment of influenza B immunity remains challenging because conventional HAI assays may exhibit lower sensitivity for detecting influenza B-specific antibodies, potentially complicating comparisons of immunogenicity across vaccine platforms and influenza subtypes [149].

### 4.6. Mucosal Immunity Induction

Another important challenge associated with mRNA vaccines is their limited ability to induce robust and durable mucosal immunity [150]. Effective protection against respiratory viruses depends, in part, on immune responses at the site of viral entry, including locally produced secretory IgA (sIgA) and tissue-resident memory T (TRM) cells within the respiratory mucosa [151]. Although intramuscular vaccination can induce high levels of circulating antibodies, it appears less effective at establishing sustained mucosal TRM cells and locally produced IgA in the respiratory tract [151,152]. Clinical studies of COVID-19 booster vaccination have further suggested that post-vaccination antibody activity in saliva may derive predominantly from the transudation of systemic IgG rather than from robust local production of secretory IgA [152]. Such serum-derived mucosal antibody responses may provide limited neutralization of antigenically divergent variants, including Omicron BA.1, at the site of viral entry [152].

Addressing this limitation may require alternative delivery strategies capable of targeting the respiratory mucosa directly. Potential approaches include intranasal administration using specialized lipid nanoparticles or other delivery systems, including plant-derived extracellular vesicles, to promote stronger local IgA responses and the establishment of mucosal TRM cells [150,151]. Such strategies could potentially complement the strong systemic immunity elicited by conventional intramuscular mRNA vaccination and improve protection at the primary site of respiratory virus infection.

## 5. Study Limitations

Some limitations should be considered when interpreting the findings of this review. First, the clinical development landscape for mRNA influenza vaccines is evolving rapidly, and a substantial number of completed or ongoing clinical trials have not yet reported their findings in peer-reviewed publications. Consequently, this review relies on information available from clinical trial registries and publicly accessible communications from vaccine developers. Such sources may provide less detailed information than full peer-reviewed publications and may be subject to subsequent updates or changes as final analyses become available.

Second, direct comparison of different mRNA influenza vaccine candidates remains challenging because clinical studies differ substantially in study design, participant characteristics, age groups, vaccine formulations, dosing regimens, comparator vaccines, influenza strains, follow-up periods and reported endpoints. In particular, differences in immunogenicity assays and definitions of serological response can limit the validity of cross-trial comparisons. Therefore, apparent differences between vaccine candidates should not be interpreted as evidence of comparative superiority unless supported by appropriately designed head-to-head studies.

Third, much of the currently available clinical evidence is based on immunogenicity and safety outcomes rather than efficacy results. Although immunogenicity measures provide important evidence of vaccine activity, they do not necessarily predict clinical effectiveness under real-world conditions. This is particularly relevant for newer mRNA platforms, for which long-term effectiveness, durability of protection and performance across different influenza seasons remain insufficiently characterized.

Finally, the rapidly changing development status of individual candidates represents an additional limitation. Trial results may become available after the completion of the present review, and some candidates may subsequently progress, be discontinued, or undergo changes in formulation or development strategy. Accordingly, the conclusions of this review should be regarded as a synthesis of the evidence available at the time of the literature search rather than a definitive assessment of the ultimate clinical role of mRNA influenza vaccines.

## 6. Comparison with Currently Available Influenza Vaccine Platforms

The potential advantages of mRNA influenza vaccines should be considered in the context of the diverse vaccine platforms that are already available for seasonal influenza prevention. Conventional egg-based vaccines remain widely used and have an extensive clinical and post-marketing evidence base, but their production is relatively time-consuming, and the use of embryonated eggs may introduce opportunities for egg-adaptive changes that can affect antigenic similarity to circulating viruses. Cell-based vaccines can reduce some of the limitations associated with egg propagation and provide an alternative manufacturing platform, although their production infrastructure is more complex and their availability remains more limited in some settings. Recombinant influenza vaccines, which do not require propagation of influenza viruses, offer advantages in manufacturing flexibility and avoid egg-adaptation-related changes. However, they require specialized production technologies and have not replaced conventional platforms in routine vaccination programs.

Formulation-based approaches have also been developed to improve immune responses in populations at increased risk of severe influenza. Adjuvanted vaccines enhance innate immune stimulation and may improve immunogenicity, particularly in older adults, whereas high-dose vaccines contain a substantially greater amount of antigen and are designed to overcome some of the age-associated reduction in immune responsiveness. These approaches therefore address limitations of conventional vaccines through mechanisms that differ fundamentally from those of mRNA vaccines.

In comparison, mRNA vaccines offer several potential platform-level advantages, including rapid redesign and manufacturing in response to antigenic changes, the ability to encode multiple influenza antigens within a single formulation and the possibility of incorporating conserved or otherwise difficult-to-produce antigens. mRNA vaccination also avoids the need for egg- or cell-based viral propagation during antigen production. However, these theoretical and technological advantages should be distinguished from demonstrated clinical benefits. Direct head-to-head evidence comparing investigational mRNA influenza vaccines with the full range of established influenza vaccine platforms remains limited. Comparisons are further complicated by differences in age groups, influenza seasons, circulating strains, antigenic match, vaccine formulations and study endpoints. Consequently, the principal advantages of mRNA influenza vaccines should currently be regarded as promising technological and manufacturing characteristics that require confirmation through adequately powered comparative clinical and real-world effectiveness studies.

## 7. Conclusions

A comprehensive analysis of the active clinical trial landscape demonstrates that mRNA technology holds substantial potential to transform influenza immunization. The growing pipeline of seasonal influenza candidates provides the foundation for expanding vaccine coverage across multiple viral targets, including through combined HA and NA formulations and multicomponent vaccines targeting overlapping respiratory threats such as SARS-CoV-2 and RSV. In parallel the development of targeted H5N1 candidates highlights the potential of mRNA platforms to support rapid pandemic preparedness and response. Emerging saRNA approaches may further enhance these applications by sustaining antigen expression, potentially enabling lower doses and more flexible dosing strategies.

Despite remaining challenges related to vaccine durability, manufacturing, distribution, and public acceptance, the candidates examined in this review collectively demonstrate a promising pathway toward broader and more adaptable influenza prevention. Ultimately, continued advances in mRNA and saRNA technologies could contribute to the development of more comprehensive multivalent vaccines and, in the longer term, support progress toward a universal influenza vaccine.

Overall, the current evidence supports a distinction between the technological potential of mRNA influenza vaccines and their clinically established benefits. Robust humoral immunogenicity and the feasibility of multivalent antigen expression have been demonstrated in clinical studies, providing clinical support for some of the proposed advantages of the platform. In contrast, several other advantages remain incompletely established. These include superior protection against influenza B, enhanced immunity in older adults, meaningful mucosal protection, broader and more durable protection against antigenically diverse viruses, and superior effectiveness compared with established egg-based, cell-based, recombinant, adjuvanted, or high-dose vaccines. Similarly, the ability to rapidly redesign mRNA vaccines represents an important technological advantage, but whether this flexibility translates into consistently improved population-level protection depends on manufacturing timelines, regulatory processes, strain selection, and antigenic matching. Most importantly, evidence demonstrating superior real-world effectiveness or clinical outcomes compared with existing influenza vaccine platforms remains limited. Thus, mRNA technology should currently be viewed as a promising platform with several clinically supported attributes, alongside a number of important advantages that remain under investigation rather than as a platform with established clinical superiority.

## Figures and Tables

**Table 1:** mRNA-based seasonal influenza vaccines evaluated clinically in adults by manufacturer and development stage.

Vaccines	Developers	Trial ID	Phase	Study Size (N)	Study Population (YoA)	Objective	Start Year	Status	Publications
Centi-flu 01	Centivax	ACTRN12626000259347	Phase 1a/b	180	18–64 ≥65	Safety and immunogenicity	2025	Recruiting	--
CVSQIV	CureVac	NCT05252338	Phase 1	240	18–55 ≥65	Safety, reactogenicity and immunogenicity	2022	Completed	--
GSK4382276A	GSK	NCT05446740	Phase 1	324	18–45 60–80	Safety, reactogenicity and immunogenicity	2022	Completed	[46]
GSK4382276A	GSK	NCT05823974	Phase 1/2	1275	18–64 65–85	Safety, reactogenicity and immunogenicity	2023	Completed	--
GSK4382276A	GSK	NCT06431607	Phase 2a	845	18–64 65–85	Safety and immunogenicity	2024	Completed	--
mRNA Flu	GSK	NCT07121192	Phase 2a	776	≥18	Safety and immunogenicity	2025	Completed	--
mRNA multivalent Flu	GSK	NCT07204964	Phase 2a	971	≥18	Safety and immunogenicity	2025	Active	--
mRNA-1010	ModernaTX	NCT05397223	Phase 1	308	18–75	Safety, reactogenicity and immunogenicity	2022	Completed	[19,20,21]
mRNA-1010	ModernaTX	NCT04956575	Phase 1/2	885	≥18	Safety, reactogenicity and immunogenicity	2021	Completed	[23,24]
mRNA-1010	ModernaTX	NCT05868382	Phase 2	270	18–49	Safety, reactogenicity and immunogenicity	2023	Completed	--
mRNA-1010	ModernaTX	NCT05606965	Phase 2	172	18–50 65–85	Safety, reactogenicity and immunogenicity	2022	Completed	--
mRNA-1010	ModernaTX	NCT05566639	Phase 3	22,502	≥50	Safety and efficacy	2022	Completed	[30]
mRNA-1010	ModernaTX	NCT05415462	Phase 3	6102	≥18	Safety and immunogenicity	2022	Completed	[30]
mRNA-1010	ModernaTX	NCT05827978	Phase 3	8411	≥18	Safety, reactogenicity and immunogenicity	2023	Completed	[33]
mRNA-1010	ModernaTX	NCT06602024	Phase 3	40,817	≥50	Safety, tolerability and immunogenicity	2024	Completed	[28]
mRNA-1011.1 mRNA-1011.2 mRNA-1012.1	ModernaTX	NCT05827068	Phase 1/2	698	50–75	Safety, reactogenicity and immunogenicity	2023	Completed	[40]
mRNA-1020 mRNA-1030	ModernaTX	NCT05333289	Phase 1/2	572	18–75	Safety, reactogenicity and immunogenicity	2022	Completed	[42]
VRC H1ssF-3928	NIAID	NCT05755620	Phase 1	50	18–49	Safety and immunogenicity	2023	Completed	--
DCVC H1 HA mRNA	NIAID	NCT05945485	Phase 1	50	18–49	Safety and immunogenicity	2023	Completed	--
cH15/3 cH4/3 cH5/1N1 cH8/1N1	NIAID	NCT07703514	Phase 1	60	18–59	Safety and immunogenicity	2026	Not yet recruiting	--
bIRV mIRV qIRV	Pfizer	NCT05052697	Phase 1/2	1158	65–85	Safety, tolerability and immunogenicity	2021	Completed	[58]
qIRV	Pfizer	NCT05540522	Phase 3	45,789	18–64 ≥65	Efficacy, safety, tolerability and immunogenicity	2022	Completed	[60]
modRNA	Pfizer	NCT06436703	Phase 1/2	1202	≥18	Safety, tolerability and immunogenicity	2024	Completed	--
modRNA	Pfizer-hVIVO	ISRCTN13789612	Phase 2a	285	18–55	Efficacy and safety	2022	Completed	[63]
modRNA	Pfizer-hVIVO	ISRCTN13559330	Phase 2a	279	18–55	Efficacy, safety and immunogenicity	2023	Completed	--
6 mRNA candidates (#1–#6)	Pfizer	NCT07431853	Phase 2	770	≥18	Safety, tolerability and immunogenicity	2026	Recruiting	--
Influenza HA mRNA	Sanofi	NCT06118151	Phase 1	388	≥18	Safety and immunogenicity	2022	Completed	--
mRNA NA	Sanofi	NCT05426174	Phase 1	233	18–64 ≥65	Safety and immunogenicity	2022	Completed	--
MRT5407	Sanofi	NCT05553301	Phase 1/2	560	≥18	Safety and immunogenicity	2022	Completed	--
MRT5410	Sanofi	NCT05624606	Phase 1/2	682	≥18	Safety and immunogenicity	2022	Completed	--
MRT5413	Sanofi	NCT05650554	Phase 1/2	682	≥18	Safety and immunogenicity	2022	Completed	--
MRT5421 MRT5424 MRT5429	Sanofi	NCT06361875	Phase 1/2	910	≥18	Safety and immunogenicity	2024	Completed	--
H3 mRNA/LNP	Sanofi	NCT05829356	Phase 1	159	18–49	Safety and immunogenicity	2023	Completed	--
Hexavalent influenza (TIV) HA mRNA + (TIV) NA mRNA	Sanofi	NCT06744205	Phase 1/2	1162	≥50	Safety and immunogenicity	2025	Completed	--

Note: The enrollment number corresponds to the value reported as “Enrollment (Actual)” in the ClinicalTrials.gov entry. --: Indicates that no peer-reviewed publication associated with the clinical trial is currently available. HA, hemagglutinin; mRNA, messenger RNA; NA, neuraminidase; NIAID, National Institute of Allergy and Infectious Diseases; qIRV, quadrivalent influenza modRNA vaccine; TIV, Trivalent; YoA: years of age.

**Table 2:** mRNA vaccine candidates against avian influenza viruses with pandemic potential, by manufacturer and development stage.

Candidate	Developers	Trial ID	Phase	Study Size (N)	Study Population (YoA)	Virus	Objective	Start Year	Status	Publications
Flu Pandemic mRNA	GSK	NCT06382311	Phase 1/2	991	18–85	Pandemic influenza (H5N1)	Safety, reactogenicity and immunogenicity	2024	Completed	--
VAL-506440	ModernaTX	NCT03076385	Phase 1	201	18–64	Influenza H10N8	Safety, tolerability and immunogenicity	2015	Completed	[81]
VAL-339851	ModernaTX	NCT03345043	Phase 1	156	18–49	Influenza H7N9	Safety and immunogenicity	2016	Completed	[81]
mRNA-1018	ModernaTX	NCT05972174	Phase 1/2	1504	≥18	Pandemic influenza (H5N8, H7N9, H5 only, and H7 only)	Safety, reactogenicity and immunogenicity	2023	Completed	[85,86]
mRNA-1018-H5	ModernaTX	NCT07496450	Phase 3	4045	≥18	Pandemic influenza (H5)	Safety, reactogenicity and immunogenicity	2026	Active	--
H5 AC-Anhui RNA H5 Astrakhan RNA	NIAID	NCT07019883	Phase 1	80	18–49	Influenza A (H5)	Safety and immunogenicity	2025	Completed	--
pdmFlu	Pfizer	NCT06179446	Phase 1	157	18–49 65–84	Pandemic influenza	Safety, tolerability and immunogenicity	2023	Completed	[89]
Pandemic Flu H5 mRNA	Sanofi	NCT06727058	Phase 1/2	276	≥18	Pandemic influenza (H5)	Safety and immunogenicity	2024	Completed	--
Pandemic flu H5 HA mRNA SD2	Sanofi	NCT06907511	Phase 1/2	720	18–64 ≥65	Pandemic influenza (H5N1)	Safety and immunogenicity	2025	Active	--

Note: The enrollment number corresponds to the value reported as “Enrollment (Actual)” in the ClinicalTrials.gov entry. YoA: years of age. --: Indicates that no peer-reviewed publication associated with the clinical trial is currently available.

**Table 3:** Vaccine candidate combinations against multiple respiratory viruses.

Candidate	Developers	Trial ID	Phase	Study Size (N)	Virus	Study Population (YoA)	Objective	Start Year	Status	Publications
mRNA-1045 mRNA-1230	ModernaTX	NCT05585632	Phase 1	392	Influenza RSV SARS-CoV-2	50–75	Safety, reactogenicity and immunogenicity	2022	Completed	--
mRNA-1073	ModernaTX	NCT05375838	Phase 1	550	Influenza SARS-CoV-2	18–75	Safety, reactogenicity and immunogenicity	2022	Completed	[99]
mRNA-1083	ModernaTX	NCT05827926	Phase 1/2	1758	Seasonal influenza SARS-CoV-2	18–50	Safety and reactogenicity	2023	Completed	[101]
mRNA-1083	ModernaTX	NCT06508320	Phase 2	932	Influenza SARS-CoV-2	50–65	Safety, reactogenicity and immunogenicity	2024	Completed	--
mRNA-1083	ModernaTX	NCT06864143	Phase 2	1320	Seasonal influenza SARS-CoV-2	18–65	Safety, reactogenicity and immunogenicity	2025	Completed	--
mRNA-1083	ModernaTX	NCT06097273	Phase 3	8061	Influenza SARS-CoV-2	50–65 ≥65	Safety, reactogenicity and immunogenicity	2023	Completed	[107,108]
mRNA-1083	ModernaTX	NCT06694389	Phase 3	2457	Influenza SARS-CoV-2	≥50	Safety, reactogenicity and immunogenicity	2025	Completed	--
qIRV/bivalent BNT162b2	Pfizer-BioNTech	NCT05596734	Phase 2	1019	Influenza SARS-CoV-2	18–64 ≥65	Safety, reactogenicity and immunogenicity	2022	Completed	--
RSVpreF + qIRV	Pfizer	NCT05788237	Phase 1	508	Influenza RSV	≥50	Safety, reactogenicity and immunogenicity	2023	Completed	--
Combined influenza and COVID-19 vaccine	Pfizer-BioNTech	NCT06683352	Phase 1/2	1353	Influenza SARS-CoV-2	≥18	Safety, tolerability and immunogenicity	2024	Completed	--
Combined influenza and COVID-19 vaccine	Pfizer-BioNTech	NCT06821061	Phase 1/2	2650	Influenza SARS-CoV-2	≥18	Safety, tolerability and immunogenicity	2025	Completed	--
Combined influenza and COVID-19 vaccine	Pfizer-BioNTech	NCT06178991	Phase 3	8795	Influenza SARS-CoV-2	18–64	Safety, tolerability and immunogenicity	2023	Completed	[113]
mRNA Flu/COVID-19	GSK	NCT06680375	Phase 1	106	Seasonal influenza COVID-19	18–64	Safety, reactogenicity and immunogenicity	2024	Completed	--
mRNA Flu/COVID-19	GSK	NCT07464314	Phase 1	226	Seasonal influenza SARS-CoV-2	65–85	Safety and immunogenicity	2026	Recruiting	--

Note: The enrollment number corresponds to the value reported as “Enrollment (Actual)” in the ClinicalTrials.gov entry. RSV: respiratory syncytial virus; YoA: years of age. --: Indicates that no peer-reviewed publication associated with the clinical trial is currently available.

**Table 4:** saRNA vaccine candidates in clinical trials in healthy adults.

Candidate	Developers	Trial ID	Phase	Study Size (N)	Virus	Study Population (YoA)	Objective	Start Year	Status
saRNA	Pfizer	NCT05227001	Phase 1	440	Influenza	18–49	Safety and tolerability	2022	Completed
saRNA	Pfizer-hVIVO	ISRCTN13789612	Phase 1/2	120	Seasonal influenza	18–55	Safety and efficacy	2022	Completed
sa mRNA influenza vaccine	Seqirus	NCT06028347	Phase 1	96	Influenza (H5N1)	18–49 65–85	Safety, reactogenicity and immunogenicity	2023	Completed
ARCT-2138	Acturus Therapeutics	NCT06125691	Phase 1	139	Seasonal influenza	18–80	Safety, reactogenicity and immunogenicity	2024	Completed
ARCT-2304	Acturus Therapeutics	NCT06602531	Phase 1	212	Pandemic influenza (H5N1)	18–85	Safety, Reactogenicity, and Immunogenicity	2024	Completed

Note: The enrollment number corresponds to the value reported as “Enrollment (Actual)” in the ClinicalTrials.gov entry. saRNA: self-amplifying RNA; YoA: years of age.

## Data Availability

No new data were created or analyzed in this study. Data sharing is not applicable to this article.
